# Immune selection during tumor checkpoint inhibition therapy paves way for NK-cell “missing self” recognition

**DOI:** 10.1007/s00251-017-1011-9

**Published:** 2017-07-11

**Authors:** Karl-Johan Malmberg, Ebba Sohlberg, Jodie P. Goodridge, Hans-Gustaf Ljunggren

**Affiliations:** 1 Department of Cancer Immunology, Institute for Cancer Research, Oslo University Hospital, Oslo, Norway; 2 The KG Jebsen Centre for Cancer Immunotherapy, Institute of Clinical Medicine, University of Oslo, Oslo, Norway; 3 Center for Infectious Medicine, Department of Medicine Huddinge, Karolinska Institutet, Karolinska University Hospital, Stockholm, Sweden; 4 Cell Therapy Institute, Nova Southeastern University, Ft Lauderdale, FL USA

**Keywords:** Natural killer (NK) cell, Education, Differentiation, Killer cell immunoglobulin-like receptors (KIR), Missing self, Tumor, Leukemia, MHC class I, HLA, Immune escape

## Abstract

The ability of NK cells to specifically recognize cells lacking expression of self-MHC class I molecules was discovered over 30 years ago. It provided the foundation for the “missing self” hypothesis. Research in the two past decades has contributed to a detailed understanding of the molecular mechanisms that determine the specificity and strength of NK cell-mediated “missing self” responses to tumor cells. However, in light of the recent remarkable breakthroughs in clinical cancer immunotherapy, the cytolytic potential of NK cells still remains largely untapped in clinical settings. There is abundant evidence demonstrating partial or complete loss of HLA class I expression in a wide spectrum of human tumor types. Such loss may result from immune selection of escape variants by tumor-specific CD8 T cells and has more recently also been linked to acquired resistance to checkpoint inhibition therapy. In the present review, we discuss the early predictions of the “missing self” hypothesis, its molecular basis and outline the potential for NK cell-based adoptive immunotherapy to convert checkpoint inhibitor therapy-resistant patients into clinical responders.

## The “missing self” hypothesis and its predictions

More than half a century ago, it was observed that F_1_-hybrid mice (derived from a cross of two inbred strains) could reject bone marrow and tumor cell grafts of parental strain-origin (Cudkowicz and Stimpfling 1964). This “F_1_-hybrid-resistance” phenomenon contrasted with the common laws of transplantation, stating that graft rejection should only takes place if the bone marrow or tumor graft carried “transplantation antigens,” e.g., foreign major histocompatibility complex (MHC) class I antigens. In the F_1_-hybrid anti-parental setting, however, no such foreign antigens existed and the rejection remained a mystery until the discovery of NK cells in the mid-1970s (Kiessling et al. 1975). It turned out that the F_1_-hybrid-resistance phenomenon could be linked to these cells (Kiessling et al. 1977). The biology of NK cell-mediated F_1_-hybrid-resistance, and related phenomena, stimulated Klas Kärre to formulate the “missing self” hypothesis for NK cell recognition of target cells (Karre 2008; Karre et al. 1986; Ljunggren and Karre 1990). The hypothesis postulated that absence, or reduced expression, of “self” MHC class I molecules could suffice to render a target cell susceptible to killing by NK cells. Noteworthy, the model explained the “F_1_-hybrid-resistance” phenomenon. However, it required experimental verification to gain acceptance.

To more directly test the predictions of the “missing self” hypothesis, the strategy was taken to select MHC class I-deficient mutant cell lines from mutagenized MHC class I-expressing (NK cell-resistant) T cell lymphoma cell lines, and to test their susceptibility to NK cell lysis. As hypothesized, such MHC class I-negative mutants were rendered sensitive to NK cell lysis in vitro and were rejected upon inoculation in immunocompetent syngeneic C57BL/6 (B6) mice (Karre et al. 1986; Ljunggren and Karre 1985). Subsequent studies demonstrated that the rejection was dependent on NK cells, in line with the earlier predictions (Karre et al. 1986; Ljunggren and Karre 1985; Ljunggren et al. 1988a, b). Experiments in which MHC class I expression was restored in the mutant cell lines subsequently confirmed that the NK cell-sensitive phenotype of the mutant cell lines was linked directly to the loss of MHC class I expression (Glas et al. 1992; Ljunggren et al. 1989, 1990b).

Two lines of research further confirmed the notion of MHC class I interference with NK cell recognition of target cells. The first was through the generation of β_2_m-deficient mice, which made it possible to study NK cell recognition of normal (i.e., untransformed) cells lacking expression of MHC class I molecules. LPS- and Con A-induced blasts from these mice were found to be susceptible to lysis in vitro by NK cells from corresponding wild type mice, and MHC class I-deficient bone marrow grafts from these mice were rejected by wild-type mice in vivo (Hoglund et al. 1991; Liao et al. 1991). The second line of research was the identification of MHC class I-specific inhibitory receptors (Karlhofer et al. 1992; Moretta et al. 1994). The generation of monoclonal antibodies against these MHC class I-binding receptors on human and murine NK cells made it possible to test critical predictions of the “missing self” hypothesis (Ljunggren and Karre 1990). One key prediction was that an inhibitory receptor-blockade should lead to augmented killing of MHC class I expressing target cells, which indeed was observed (Karlhofer et al. 1992; Moretta et al. 1994).

While the identification of inhibitory receptors in part uncovered the molecular mechanisms used by NK cells to recognize MHC class I deficient tumor cells, later studies showed that sensing the absence of self-MHC class I molecules is not sufficient to cause target cell killing. NK cells also need stimulation by target cell ligands to trigger activation via specific receptors. The identity of the latter (Bauer et al. 1999; Pessino et al. 1998; Vitale et al. 1998) remained elusive until several years after the discovery of inhibitory receptors. We now know that NK cell recognition of tumor and bone marrow grafts is tightly regulated by processes involving the integration of signals delivered from multiple activating and inhibitory receptors (Lanier 2005). The nature and molecular specificity of the key inhibitory receptors are described in the following section.

## The molecular basis for “missing self” reactivity

NK cell function, including the ability to sense “missing self” MHC class I molecules, is determined by the integrated signaling through multiple activating and inhibitory receptors (Bryceson et al. 2006; Lanier 2005). Mice and humans use two structurally unrelated receptor families; the Ly49 and KIR receptors, respectively, to recognize MHC class I molecules (Parham 2005b). Although the two receptor families have evolved independently, they share many features including a stochastic expression pattern and a central role in the formation of a functional diversification within the NK cell repertoire (Parham 2008).

The human *KIR* gene cluster is located within the leukocyte receptor complex on chromosome 19 (Wende et al. 1999) and displays an extensive diversity between individuals. Generally, *KIR* haplotypes contain between 9 and 17 genes (15 genes according to the most recent update of the nomenclature (Personal communication, Steven Marsh, Anthony Nolan Research Institute, UK) (Uhrberg et al. 1997, 2002), although studies of gene copy number variations have revealed a *KIR* haplotype with only four genes (Traherne et al. 2010). Additionally, KIR genes contain variable sites, which result in multi-allelic polymorphism (Gardiner et al. 2001; Shilling et al. 2002; Uhrberg et al. 1997; Wagtmann et al. 1995). Hence, it is very unlikely that two randomly selected individuals share the same *KIR* genotype (Shilling et al. 2002).

Four major inhibitory KIRs have been identified (KIR2DL2/3, KIR2DL1, KIR3DL1, and KIR3DL2) that recognize polymorphic residues within the α1 and α2 domains of the HLA class I heavy chain. KIR2DL2/3 recognize HLA-C allotypes with asparagine 80 (C1), KIR2DL1 recognizes HLA-C allotypes with lysine 80 (C2), KIR3DL1 recognizes HLA-A and HLA-B allotypes with Bw4 motifs at positions 77–83, and KIR3DL2 recognizes HLA-A3/11 and HLA-F (Goodridge et al. 2013; Parham 2005a). The activating receptors KIR2DS1, KIR2DS2, and KIR3DS1 display similar extracellular domains as their inhibitory counterparts and are thus thought to share binding specificities. KIR2DS1 and KIR2DS2 have shown to weakly bind to HLA-C2 and HLA-C1, respectively (Biassoni et al. 1997; Stewart et al. 2005); however, interaction between KIR3DS1 and Bw4 has not been confirmed. KIR2DS4 is believed to recognize HLA-C*1601 (C1), HLA-C*0501 (C2), and HLA-A*1102 (Graef et al. 2009). To date, KIR2DS3, KIR2DS5, and KIR2DL5 have no known ligands.

A unique feature of KIR and Ly49 receptors is their stochastic distribution across the NK cell population (Anderson 2006; Uhrberg 2005). In combination with genetic variability of the *KIR/Ly49* locus at the levels of gene content, copy number variation, and allelic polymorphism, the stochastic expression of the *KIR* gene products results in highly diverse NK cell-repertoires among individuals. Unlike the positive and negative selection of T cells in the thymus, there is no intrinsic selection process that delete NK cells that lack inhibitory receptors to self HLA class I (Andersson et al. 2009). However, most KIR/Ly49-negative NK cells express CD94/NKG2A, an inhibitory receptor that binds to the ubiquitously expressed HLA-E molecule (in humans) and Qa1 (in mice) (Andersson et al. 2009; Braud et al. 1998; Vance et al. 1998; Veinotte et al. 2003; Yawata et al. 2008). The inverse correlation between strong KIR-HLA alleles and HLA alleles that favor functional NKG2A/HLA-E interactions is a striking example of complementary evolution (Horowitz et al. 2016). The intuitive function of these complementary inhibitory receptor-ligand pairs is to preserve tolerance to self by limiting autoreactivity against tissues with expression of self-HLA class I. The expression of self-inhibitory receptors is tightly linked to the ability of NK cells to gain functional potential during a process termed education (Anfossi et al. 2006). Consequently, NK cells that lack self-specific inhibitory receptors, approximately 10% of all NK cells in both mice and humans, are hyporesponsive (Fauriat et al. 2008; Fernandez et al. 2005). In the next section, we discuss how this functional calibration against self-MHC class I allows NK cells to sense the loss of MHC class I on target cells.

## Calibration of “missing self” reactivity through MHC class I recognition

In recent years, it has become clear that inhibitory receptors not only abrogate functional responses in NK cells during the effector phase, but also tune the cell-intrinsic functional potential during homeostatic conditions. Thus, inhibitory interactions with self-MHC class I translate into a quantitative relationship between self-recognition and effector function. Paradoxically, NK cells expressing self-MHC class I specific inhibitory receptors, receiving constitutive inhibitory input, exhibit gained functionality, including cytokine production and killing activity. This functional calibration against self-MHC class I is critical for the ability of NK cells to recognize and kill target cells displaying reduced MHC class I expression.

NK cell education can be observed at the population level by challenging the NK cells with various stimuli. All NK cell effector functions, from the most proximal Ca-flux, adhesion and formation of the immune synapse, induction and secretion of cytokines/chemokines, exocytosis of cytolytic granules all the way to in vivo killing of MHC class I-mismatched targets can be linked to the educational status of the NK cell (Anfossi et al. 2006; Guia et al. 2011; Thomas et al. 2013; Tu et al. 2014). Mouse models have demonstrated that the functional phenotype induced by education is dynamic and dependent on the net signaling input to NK cells from hematopoietic cells and to some extent from stromal cells (Ebihara et al. 2013). Previous experiments have shown that transfer of mature NK cells from one MHC class I environment to another results in reshaping of the functional potential based on the inhibitory input in the new MHC class I setting (Ebihara et al. 2013; Elliott et al. 2010). Genetic knockdown of SLAM-family receptors by CRISPR/Cas9 induces hyperfunctionality (Chen et al. 2016), whereas deletion of the inhibitory signaling through ITIM and SHP-1 renders NK cells hypofunctional (Kim et al. 2005; Viant et al. 2014). These results highlight the important role of cell-to-cell communication in the regulation of NK cell function. However, it still remains unclear how the net signaling input through activating and inhibitory receptors during education translates into a given functional potential of the cell.

The continuous calibration of the cytotoxic potential serves to maintain tolerance at steady state and determines the response of the individual cell to sudden changes in the environment (i.e., discontinuity) (Pradeu et al. 2013). According to the discontinuity theory of immunity, the immune system is geared to detect sudden changes in the host (Pradeu et al. 2013). In this context, education provides the cell with a capability of detecting discontinuity, such as the loss of MHC-class I ligands, or as we shall discuss next, to mediate alloreactivity upon adoptive transfer to a new HLA class I environment in settings of human transplantation.

## Transfer of NK cells across HLA barriers—missing the “new” self MHC class I

Transfer of NK cells across HLA class I barriers can release the cytotoxic potential of a functional repertoire that is otherwise restrained by interactions with self HLA class I molecules in the donor (Ruggeri et al. 1999; Valiante et al. 1997; Yawata et al. 2008). Hence, educated NK cells can sense “missing self” in a new HLA class I environment. This scenario occurs in certain specific donor-recipient combinations in the context of partially mismatched allogeneic hematopoietic stem cell transplantation (HSCT) and has been linked to improved survival (Giebel et al. 2003; Miller et al. 2007; Ruggeri et al. 2002; Symons et al. 2010). For such an NK cell-mediated graft-versus-leukemia (GVL) effect to take place, the recipient must lack any one of the three major KIR-ligands present in the donor.

Notably, most adoptive NK cell trials conducted until now in cancer patients have been based on transfer of polyclonal NK cells across HLA class I barriers, with the aim of eliciting reactivity towards “missing” HLA class I ligands in the new hematopoietic environment (the latter representing the “new self”). However, in many of these studies it has been difficult to attribute the clinical efficacy to the *KIR*/*HLA* genetics of the donors (Miller et al. 2005). One possible explanation for this outcome is that most currently used NK cell products contain a limited proportion of alloreactive NK cells, despite a genetically predicted KIR-HLA mismatch (Fauriat et al. 2008). Indeed, the size of the alloreactive repertoire determine the ability of allogeneic NK cells to kill mismatched tumor cells (Pende et al. 2009), and was recently shown to influence clinical outcomes in terms of induction of molecular remission and prolonged disease-free survival in AML (Curti et al. 2016). New insights into the functional diversification of NK cells hold promise for the next generation NK cell therapy based on selective expansion or directed differentiation of specific cell populations with enhanced tumor-killing capacity.

## Adaptive NK cells—“missing self” unleashed

As stated above, a variety of different NK cell-based products have been infused to patients with cancer (in particular leukemia) to achieve tumor eradication or reduction (reviewed in ref. (Knorr et al. 2014)). In this context, significant interest has been drawn more recently to the utilization of a specific subpopulation of terminally differentiated NK cells, often referred to as “adaptive” (or memory) NK cells (Liu et al. 2015). Adaptive NK cells, expressing the cell surface receptor NKG2C, are found at varying frequencies in a fraction of CMV seropositive donors. These cells can be efficiently expanded in vitro by stimulation with IL-15 together with feeder cells overexpressing HLA-E (Beziat et al. 2013). Different approaches to expand such adaptive NK cells were recently reviewed by Liu and collaborators (Liu et al. 2015). A unique feature of this NK cell subset is the expression of one single self-HLA class I specific KIR. Noteworthy, such single KIR^+^ adaptive NK cells may represent up to 75% of all NK cells in some healthy donors (Beziat et al. 2013). Thus, transfer of adaptive NK cells across HLA class I-barriers represents a way to maximize the effects of a “missing self” response. As noted, adaptive NK cells typically express NKG2C and are also negative for NKG2A and are therefore particularly attractive as effector cells also against tumor cells that overexpress HLA-E, either spontaneously or as a result of immune escape (Gooden et al. 2011). Thus, although adaptive NK cells have so far only been shown to expand in response to infection it may be possible to harness their unique properties in cell therapy settings against human cancer.

In support of the notion above, culture of polyclonal NK cells in IL-15 together with feeder cells transfected with HLA-E for 14 days led to an enrichment of adaptive NK cells with distinct KIR specificities that were determined by the HLA-C1/C2 genotype of the donor (Liu et al. 2017). The ex vivo expanded adaptive NK cells gradually obtained a more differentiated phenotype and displayed efficient killing of HLA class I-mismatched T and precursor B cell acute lymphoblastic leukemia (ALL) blasts, previously shown to be refractory to NK cell killing. Selective expansion of NK cells that express one single inhibitory KIR for self-HLA class I would allow exploitation of the full potential of the “missing self” response in cancer immunotherapy across HLA class I barriers.

**Fig. 1:**
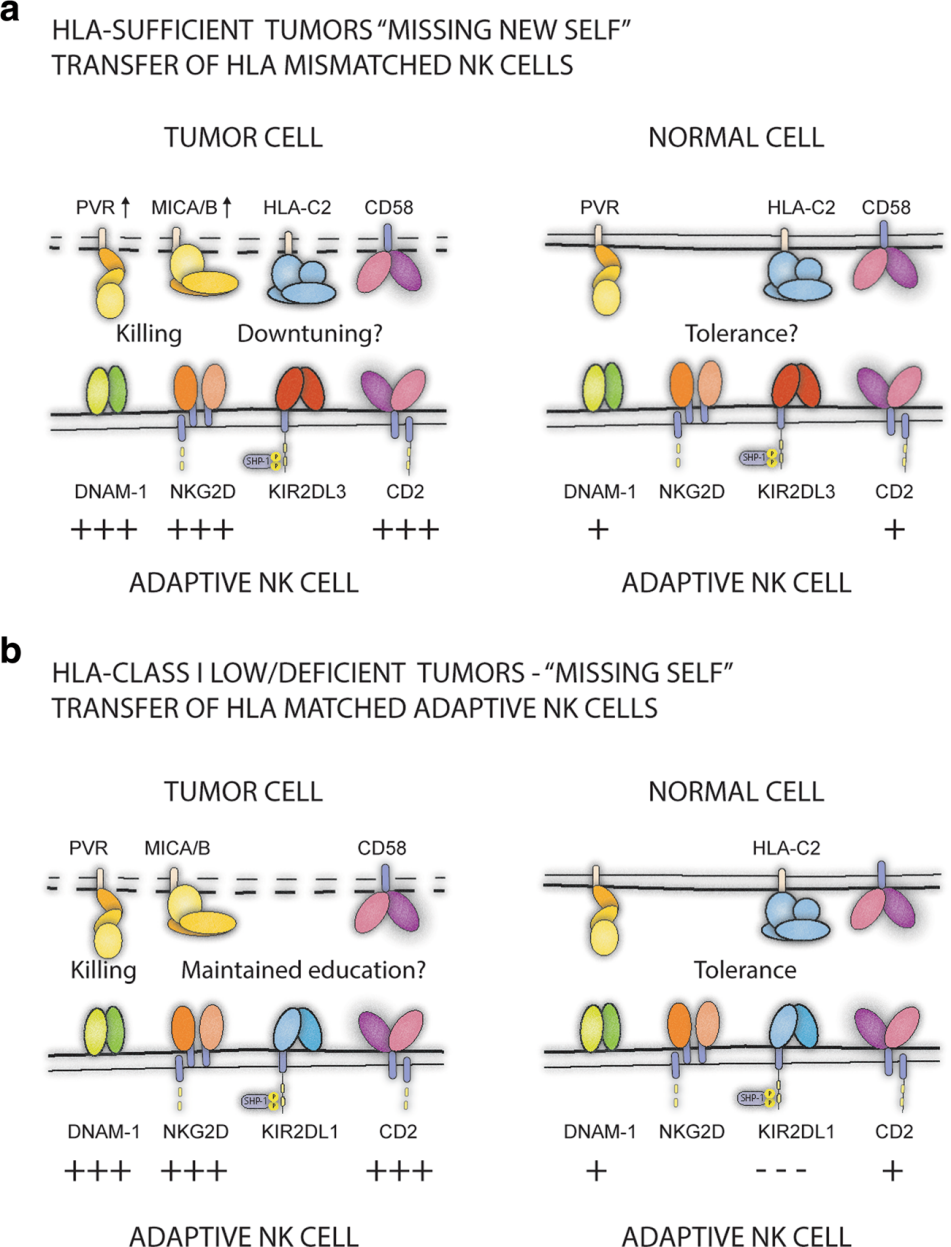
Exploiting “missing self” reactivity against HLA class I mismatched or deficient tumor targets. A schematic illustration of the conceptual basis for the usage of adaptive single-KIR^+^ NK cells in clinical cell therapy settings. **a** By selectively expanding NK cells expressing, for example, KIR2DL3 as their only inhibitory KIR, the potential for “missing self” recognition can be amplified when these cells are transferred across HLA class I barriers, to a recipient lacking the HLA-C1 molecule. The specific targeting of tumor cells relies on induced expression of ligands (PVR and MICA) binding to activating receptors DNAM-1 and NKG2D, respectively. Likewise adaptive NK cells express high levels of CD2 that may contribute to target cell recognition of cells that express CD58. It remains an open question whether effector function will be down-tuned in the new MHC class I environment due to the absence of self HLA class I on all recipient cells. **b** Adaptive NK cells can also be used against tumors displaying spontaneous or therapy-induced loss of HLA class I molecules. If autologous or HLA class I matched allogeneic NK cells are used as a source to differentiate/selectively expand the adaptive NK cell population, normal tissue will be spared. There is also the possibility of maintained education of adaptive NK cells against self HLA class I expressed by normal cells

The most immediate setting for clinical implementation is to transfer ex vivo expanded adaptive NK cells across HLA-C barriers (Fig. 1a). In such protocols, cells generated from a C1/C1 donor could be transferred to patients with C2/C2 genotypes and vice versa. Notably, C1/C2 donors could also be used pending on their CMV-imprinted repertoire. In C1/C2 donors with a pre-existing expansion of 2DL1^+^ adaptive NK cells, it could be possible to further expand such cells, extending the number of available donors for C1/C1 patients (Liu et al. 2017), which may be of clinical relevance given that the C2/C2 genotype is rather rare.

However, we foresee that adaptive NK cells may also be used to fully explore the clinical potential of “missing self” recognition in the context of partial or total HLA class I loss, either spontaneous or induced by T cell-based cancer immunotherapy. Specifically, as we shall discuss in the next section, NK cell-based strategies may hold promise as a rescue therapy for patients that acquire resistance to tumor-specific CD8 T cells, and hence also checkpoint inhibition, due to loss of HLA class I expression.

## Natural and acquired resistance to cytotoxic T cells

A consequence of immune-mediated tumor recognition is a continuous “sculpturing” of the tumor phenotype, referred to as immunoediting (Schreiber et al. 2011). The immune selection pressure favors the development of less immunogenic tumors, which escape recognition by a functioning immune system. Presentation of antigens in the context of HLA class I molecules is critical for CD8 T cell priming, and during the effector phase of an adaptive immune response. Alterations in antigen processing and presentation are commonly seen both during viral infection and in malignancies.

Viruses have adapted to the immune-mediated selection pressure in numerous ingenious ways, affecting almost every part of the MHC class I presentation pathway (Yewdell and Hill 2002). In tumors, the antigen presentation pathway can be disrupted as a consequence of mutations and/or deletions of one or several genes encoding components of the antigen presentation machinery. Complete loss of HLA class I molecules is a common event in several murine and human tumors including melanoma, diffuse large cell B cell lymphoma (DLBCL), cervical, lung, prostate, and renal cell carcinoma (Garrido et al. 2016; Khong and Restifo 2002). In fact, loss of HLA class I has been reported in up to 80% of non-small cell lung cancer and 50% of DLBCL (Bukur et al. 2012). A survey of several melanoma cell lines revealed little or no expression of genes of the HLA-C locus (Marincola et al. 1994). Since the MHC class I molecule is not properly assembled in the absence of peptide and/or β2m (Ljunggren et al. 1990a), loss of HLA class I may depend on alteration of β_2_m expression or mutations of proteasomal subunits as well as of transporters associated with antigen presentation (TAP1/2) (Seliger et al. 2002).

Tumors may also often display a selective loss of HLA-haplotype, locus or allele. Haplotype losses may be the result of loss of heterozygocity (LOH) on chromosome 6 (Ramal et al. 2000). In addition to these irreversible mechanisms underlying loss of HLA class I, there are a number of reversible (“soft”) causes of low HLA class I expression that may possibly be manipulated through immune interventions to achieve tumor rejection (Garrido et al. 2017). In DLBLC, 29% of the cases displayed mutations and deletions of the *β2m* gene (Challa-Malladi et al. 2011). However, additional soft mechanisms led to aberrant expression of HLA class I in more than 60% of all analyzed cases. Intriguingly, the gradual loss of HLA class I during tumor progression appears to be paralleled with exclusion of T cell infiltrates, suggesting a tight interplay between immune recognition and structural organization of the tumor tissue (Garrido et al. 2017).

Loss of HLA class I may also occur as a result of ongoing immunotherapy. Sequential loss of several HLA class I alleles in successive metastases from a patient undergoing immunotherapy suggests that immune mediated selection pressure plays a pivotal role in selecting HLA class I loss variants (Lehmann et al. 1995). β_2_m mutations along with LOH of the second allele have been described during adoptive immunotherapy (Restifo et al. 1996; Sucker et al. 2014). Therapy-induced loss of HLA class I due to an acquired point mutation in β2m has also been described to lead to resistance to tumor specific CD8 T cells despite retained antigen expression (Rosenberg et al. 2003).

Immunotherapy with checkpoint inhibition, such as anti-programmed death 1 (PD-1) or anti-CTLA-4, has led to a paradigm shift in cancer treatment. Tumor infiltrating CD8 T cells are the main effector cells that kill cancer cells during PD-1 blockade therapy (Tumeh et al. 2014). Most objective responses to anti-PD-1 and anti-CTLA-4 are durable (Hamid et al. 2013; Prieto et al. 2012; Schadendorf et al. 2015). However, in one study approximately 25% of patients with melanoma who had an objective response to PD-1 blockade therapy had disease progression at a median follow-up of 21 months (Ribas et al. 2016). Acquired resistance to anti-PD-1 therapy has been associated with alterations in the antigen presentation pathway (Zaretsky et al. 2016). Whole exome sequencing in four patients with relapsing metastatic melanoma revealed clonal selection and outgrowth resistant tumors with loss-of-function mutations in the genes encoding interferon-receptor-associated Janus kinase 1 (JAK1) or Janus kinase (JAK2), together with deletion of the wild-type allele in two patients (Zaretsky et al. 2016). A third patient displayed a truncating mutation in the β2m gene, associated with a complete loss of HLA class I at the surface. Although these resistance mechanisms were so far only identified in a limited number of patients, the fact that three of four patients with acquired resistance to immunotherapy displayed alterations in the antigen presentation machinery suggests that this may be a common mechanism. Furthermore, given the frequent loss of HLA class I in a wide range of tumor samples, it is tempting to speculate that alterations in the antigen presentation pathway are implicated also in the primary resistance to checkpoint inhibition. Ongoing efforts to decipher mechanisms of resistance will be helpful in determining if some of these patients may instead be targeted by NK cell-based immunotherapies.

## The search for missing self—30 years later

Since NK cells specifically sense the absence of HLA class I, adoptive NK cell therapy may be an attractive rescue option for patients that fail conventional immunotherapy or in an upfront setting for patients with tumors displaying low levels of HLA class I at diagnosis. Indeed, loss of β_2_m leads to increased NK sensitivity and effective tumor elimination by NK cells (Glas et al. 1992). Although one could theoretically use autologous NK cells in these contexts, unmanipulated autologous NK cells express highly diverse KIR repertoires and may often be functionally suppressed (Carlsten et al. 2010; Malmberg and Ljunggren 2006). An advantage of adaptive NK cells is their predictable and homogenous expression of self-KIR rendering this highly cytolytic subset completely tolerant to normal tissues (Fig. 1b). Furthermore, adaptive NK cells display potential for long-term persistence, possibly even self-renewal (Beziat et al. 2013; Corat et al. 2017; Schlums et al. 2017), and could therefore achieve long-term engraftment, even in the absence of conditioning.

Nevertheless, there are several bottlenecks that need to be addressed for the successful clinical implementation of next generation NK cell strategies. First, we have limited insights into the mechanisms that regulate homing of NK cells to the tumor. Patient-derived NK cells are rarely found in tumors and often display a naïve CD56^bright^ phenotype (Carrega et al. 2008; Del Mar Valenzuela-Membrives et al. 2016). Second, experimental models in mice suggest that NK cells down-tune their functional potential within 48 h following transfer to a new environment (Joncker et al. 2010). Third, additional genetic alterations in the tumor may impede immune cell recognition. For example, a large fraction of DLBCL cases display genetic inactivation of CD58, which is the ligand for the co-activation receptor and adhesion molecule CD2 (Challa-Malladi et al. 2011). CD2 is critical for both T and NK cell adhesion to the target and subsequent activation (Bolhuis et al. 1986; Kanner et al. 1992; Wang et al. 1999). Despite these hurdles, we believe that NK cell recognition of “missing self” is likely more clinically relevant than ever before and may hold promise to convert immune failures into clinical responders. New insights into the mechanisms that determine the dynamic tuning of NK cells during education may provide means to manipulate NK cell function to help maintain the functional potential of alloreactive NK cells in vivo. NK cells may also be engineered to express chimeric antigen receptors for enhanced targeting and/or to express adhesion molecules or homing receptors.
